# *Stem lodging Resistance-1* controls stem strength by positively regulating the biosynthesis of cell wall components in *Capsicum annuum* L.

**DOI:** 10.1093/hr/uhae169

**Published:** 2024-06-20

**Authors:** Qing Li, Canfang Fu, Bozhi Yang, Huiyang Yu, Huan He, Qing Xu, Wu Miao, Rongyun Liu, Wenchao Chen, Zhuqing Zhang, Xuexiao Zou, Bowen Hu, Lijun Ou

**Affiliations:** Engineering Research Center of Education, Ministry for Germplasm Innovation and Breeding New Varieties of Horticultural Crops, Key Laboratory for Vegetable Biology of Hunan Province, College of Horticulture, Hunan Agricultural University, Changsha 410125, China; Yuelushan Lab, Changsha 410128, China; Shenzhen Branch, Guangdong Laboratory for Lingnan Modern Agriculture, Key Laboratory of Synthetic Biology, Ministry of Agriculture and Rural Affairs, Agricultural Genomics Institute at Shenzhen, Chinese Academy of Agricultural Sciences, Shenzhen, 518120, China; Engineering Research Center of Education, Ministry for Germplasm Innovation and Breeding New Varieties of Horticultural Crops, Key Laboratory for Vegetable Biology of Hunan Province, College of Horticulture, Hunan Agricultural University, Changsha 410125, China; Yuelushan Lab, Changsha 410128, China; Engineering Research Center of Education, Ministry for Germplasm Innovation and Breeding New Varieties of Horticultural Crops, Key Laboratory for Vegetable Biology of Hunan Province, College of Horticulture, Hunan Agricultural University, Changsha 410125, China; Yuelushan Lab, Changsha 410128, China; Engineering Research Center of Education, Ministry for Germplasm Innovation and Breeding New Varieties of Horticultural Crops, Key Laboratory for Vegetable Biology of Hunan Province, College of Horticulture, Hunan Agricultural University, Changsha 410125, China; Yuelushan Lab, Changsha 410128, China; Engineering Research Center of Education, Ministry for Germplasm Innovation and Breeding New Varieties of Horticultural Crops, Key Laboratory for Vegetable Biology of Hunan Province, College of Horticulture, Hunan Agricultural University, Changsha 410125, China; Yuelushan Lab, Changsha 410128, China; Engineering Research Center of Education, Ministry for Germplasm Innovation and Breeding New Varieties of Horticultural Crops, Key Laboratory for Vegetable Biology of Hunan Province, College of Horticulture, Hunan Agricultural University, Changsha 410125, China; Yuelushan Lab, Changsha 410128, China; Hunan Xiangyan Seed Industry Co., Ltd, Changsha, 410100, China; Hunan Xiangyan Seed Industry Co., Ltd, Changsha, 410100, China; Vegetable Research Institute, Hunan Academy of Agricultural Science, Changsha, 410125, China; Vegetable Research Institute, Hunan Academy of Agricultural Science, Changsha, 410125, China; Engineering Research Center of Education, Ministry for Germplasm Innovation and Breeding New Varieties of Horticultural Crops, Key Laboratory for Vegetable Biology of Hunan Province, College of Horticulture, Hunan Agricultural University, Changsha 410125, China; Yuelushan Lab, Changsha 410128, China; Engineering Research Center of Education, Ministry for Germplasm Innovation and Breeding New Varieties of Horticultural Crops, Key Laboratory for Vegetable Biology of Hunan Province, College of Horticulture, Hunan Agricultural University, Changsha 410125, China; Yuelushan Lab, Changsha 410128, China; Engineering Research Center of Education, Ministry for Germplasm Innovation and Breeding New Varieties of Horticultural Crops, Key Laboratory for Vegetable Biology of Hunan Province, College of Horticulture, Hunan Agricultural University, Changsha 410125, China; Yuelushan Lab, Changsha 410128, China

## Abstract

Lodging presents a significant challenge in cultivating high-yield crops with extensive above-ground biomass, yet the molecular mechanisms underlying this phenomenon in the *Solanaceae* family remain largely unexplored. In this study, we identified a gene, *CaSLR1* (*Capsicum annuum Stem Lodging Resistance 1*), which encodes a MYELOBLASTOSIS (MYB) family transcription factor, from a lodging-affected *C. annuum* EMS mutant. The suppression of *CaSLR1* expression in pepper led to notable stem lodging, reduced thickness of the secondary cell wall, and decreased stem strength. A similar phenotype was observed in tomato with the knockdown of *SlMYB61*, the orthologous gene to *CaSLR1*. Further investigations demonstrated that *CaNAC6*, a gene involved in secondary cell wall (SCW) formation, is co-expressed with *CaSLR1* and acts as a positive regulator of its expression, as confirmed through yeast one-hybrid, dual-luciferase reporter assays, and electrophoretic mobility shift assays. These findings elucidate the *Ca*NAC6-*Ca*SLR1 module that contributes to lodging resistance, emphasizing the critical role of *CaSLR1* in the lodging resistance regulatory network.

## Introduction

Stems are essential for plant growth and development, as they not only facilitate the transport of water and nutrients but also provide critical structural support. Stem lodging can drastically affect crop production, significantly reducing yield and quality while increasing harvesting costs [1]. Consequently, research into stem lodging resistance (SLR) is vital for advancing sustainable agricultural practices. Many studies have shown that lodging resistance is closely related to the thickness of the secondary cell wall (SCW) [1, 2]. Cellulose, hemicellulose and lignin are the main components of the cell wall, enhancing the mechanical strength of plants [3, 4]. In the brittle stem mutant of rice, there was a notable decrease in the stem’s mechanical strength and its cellulose content [5]. Lignin is considered an important factor in lodging resistance across different varieties [6]. Enhancing the expression of cell wall structural genes like *4-coumarate: CoA ligase 3* (*4CL3*) and *phenylalanine ammonia-lyase* (*PAL*) within the lignin biosynthesis pathways bolstered cell wall thickening, thus reinforcing mechanical support in rice [2]. Therefore, stem cell wall components play critical roles in enhancing mechanical strength and maintaining stability in stems.

Previous studies have shown that secondary wall NACs (SWNs) transcription factors and MYB transcription factors (e.g., MYB46, MYB83) mediated transcription networks played key regulatory roles in plant SCW formation [7, 8]. Studies on cotton and *Arabidopsis thaliana* have showed that concurrent disruption of *secondary wall-associated NAC domain protein 1* (*SND1*) and *NAC secondary wall thickening promoter 1* (*NST1*) results in stem droop, indicating functional redundancy among NAC transcription factors in stem development [9, 10]. Notably, inhibiting *AtMYB46* alone can lead to a stem droop phenotype [11], suggesting that these MYB TFs play a crucial role in regulating stalk development. SWNs bind to specific promoter sequences, known as secondary NAC binding elements (SNBEs), in MYB46 and MYB83, thereby regulating SCW biosynthesis [12–14]. Nonetheless, our understanding of the pivotal role MYB TFs play in regulating plant cell wall development remains limited.

Pepper (*Capsicum annuum* L.) is an important vegetable crop due to its distinctive spicy components [15–17]. However, during production, pepper often faces challenges such as flower and fruit drop after stem lodging, which severely impairs pepper’s yield and quality [1]. Despite its importance, research on stem lodging resistance in peppers is notably lacking. In this study, we identified a distinct stem lodging mutant *slr1* (*stem lodging resistance 1*) through ethylmethane sulfonate (EMS) mutagenesis. Genetic analysis revealed that the*slr1* phenotype is likely governed by a recessive gene located on chromosome 8, encoding MYB61. Knock down of *CaSLR1* resulted in stem bending and lodging, a phenotype was also observed in CRISPR/Cas9-mediated knockout of *SlMYB61*, a homolog of *Ca*SLR1 in tomato. Additionally, knockdown *CaSLR1* led to the downregulation of numerous genes associated with cell wall formation, consequently causing thinner secondary cell walls and reduced stem strength. Furthermore, we identified a SCW formation-related gene, *Ca*NAC6, co-expressed with *CaSLR1*, with *Ca*NAC6 positively regulating the expression of *CaSLR1*. This suggests that *CaSLR1* may serve as a central hub in the secondary cell wall (SCW) regulatory network, enhancing stem lodging resistance in pepper. Our study provides theoretical support for understanding the molecular regulatory mechanisms in stem development. It offers the target genes for molecular designing breeding programs aimed at enhancing lodging resistance in peppers and tomatoes.

## Results

### Phenotypic characteristics of *slr1*

**Figure 1:**
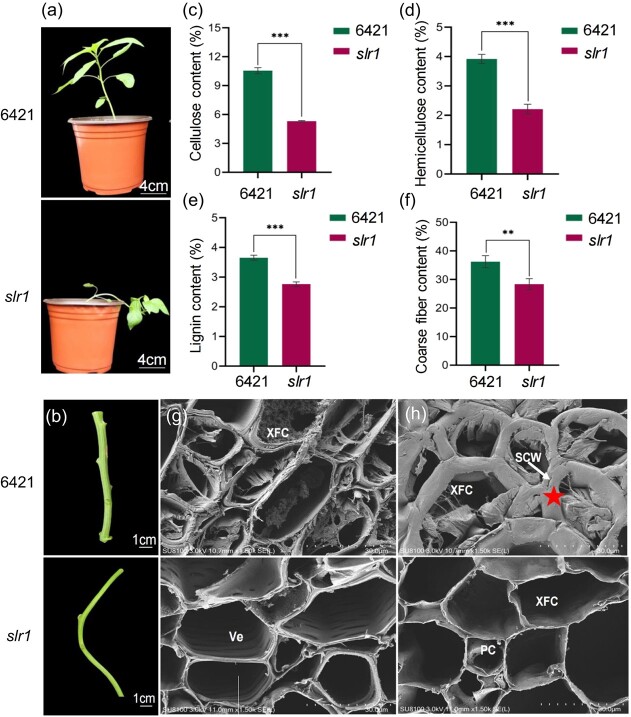
Phenotype characteristics of wild-type (WT) and *slr1* mutant plants. **(a)** Plants at the beginning of flowering, scale = 6 cm. **(b)** Stem from WT and *slr1* mutants, scale = 1 cm. **(c)** Cellulose content (%). **(d)** Hemicellulose content (%). **(e)** Lignin content (%). **(f)** Coarse fiber content (%). The results were expressed as the mean ± SE (*n* = 3). ^**^P < 0.01 and ^***^P < 0.001, as determined by Student's *t*-test. **(g)** Secondary xylem from the WT and *slr1* plants. Scale = 30 μm. XFC: xylem fiber cells; Ve: vessel. **(h)** The periphery of the secondary xylem in the WT and *slr1* plants. Scale = 30 μm. XFC: xylem fiber cell; PC: parenchyma cell. Five-pointed star showed thickened secondary XFC walls.

To explore the phenotypic characteristics of stem lodging development in *slr1*, we compared the growth parameters of WT and *slr1* mutants. At 45 days, all *slr1* mutant plants exhibited evident stem lodging and twisting compared to the WT, whereas this lodging phenomenon was not observable at 25 days of seedling growth (Fig. 1a and b; Fig. S1a, see online supplementary material). Notably, the *slr1*mutant exhibited abnormal reproductive development with delayed first flower and fruit set, accompanied by a significant reduction in the number of fruits and germination rate (Fig. S1b–d, see online supplementary material). Furthermore, we compared the growth differences between 45-day-old WT and *slr1* mutants. Compared to WT, the aboveground, root, total fresh weights, and first internode length of *slr1* mutants were significantly reduced by 33.38, 40.82, 37.11, and 50%, respectively (Fig. S1e–h, see online supplementary material). However, no significant difference was observed in the leaf length-width ratio (Fig. S1i, see online supplementary material). Since cellulose, hemicellulose, and lignin are critical components in lodging resistance [3, 4], the contents of cell components in the stems of WT and *slr1* were measured. Compared to WT, the cellulose, hemicellulose, lignin, and crude fiber contents of *slr1* mutants significantly decreased by 49.75, 36.81, 24.36, and 21.89%, respectively (Fig. 1c–f).

In addition, SEM was performed to compare the difference in stem structure in WT and *slr1* mutant. The results revealed that the hollow vessels within the xylem of the *slr1 mutant was* obvious presence*,* whereas the WT exhibited more xylem fiber cells (XFCs) (Fig. 1g). Conversely, in the WT, XFCs with thickened SCWs were observed at the periphery of secondary xylem, while parenchyma-like cells (PCs) were prevalent in the corresponding tissues of *slr1* (Fig. 1h). These findings suggest that the abnormal development of xylem led to stem lodging in the *slr1* mutant.

### Identification of the target gene in *slr1* mutant

**Figure 2:**
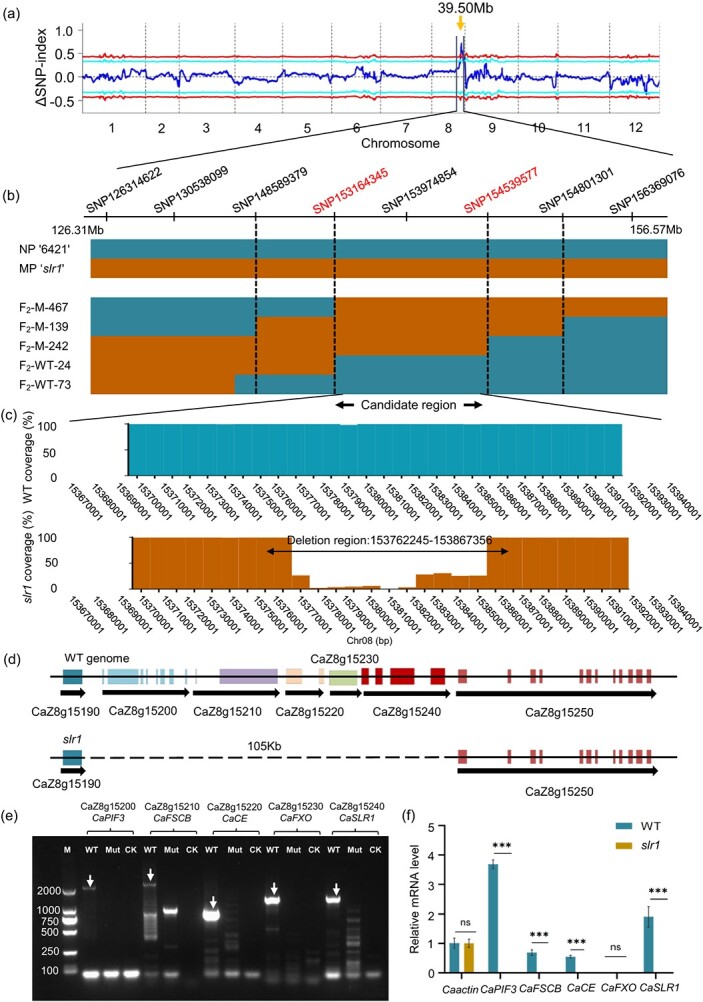
Mapping of the genes related to stem lodging using Mutmap. **(a)** Mutmap indicated a preliminary map region. The candidate chromosomal region of about 39.50 Mb was marked in a arrow. **(b)** Candidate regions were narrowed down by genotyping. NP, WT chromosomal . MP, *slr1* chromosomal. The normal phenotype individual plants with chromosomal exchanges in the F_2_ population., including WT-24 and WT-73. The chromosomal exchanges plants with a lodging phenotype in the F_2_ population, were marked M-467, M-139, and M-242. The candidate region was identified as the chromosomal domain between the two SNP markers (SNP153164345 and SNP15453957). **(c)** Analysis of the chromosomal structural variation within the range of 153.16–154.53 Mb. Vertical axis: Depth coverage rate between WT and *slr1*; Abscissa axis: chromosome position. **(d)** Approximately 105 Kb was deleted in the genome of *slr1* mutant, including CaZ8g15200, CaZ8g15210, CaZ8g15220, CaZ8g15230, and CaZ8g15240. **(e)** The agarose gel electrophoresis was conducted using PCR products of five genes from both WT and *slr1* plants. The genes and their respective PCR product sizes are as follows: CaZ8g15200 (*CaPIF3*, 2181 bp); CaZ8g15210 (*CaFSCB*, 2385 bp); CaZ8g15220 (*CaCE*, 810 bp); CaZ8g15230 (*CaFXO*, 1098 bp); and CaZ8g15240 (*CaSLR1*, 1263 bp). **(f)** The expression levels of the five genes in the stems of the WT and *slr1* plants. The results are presented as mean ± SE (*n* = 3). ^*^P < 0.05, ^**^P < 0.01, ^***^P < 0.001, and n.s, not significant, as determined by the Student's *t*-test.

To map the target genes associated with stem lodging, we created an F_2_ segregating population *with slr1* as the female parent and WT as the male parent for crossbreeding. We obtained a total of 483 F_2_ individuals, comprising 402 wild-type phenotypes and 81 mutant phenotypes. The Chi-square test revealed that the F_2_ population significantly deviated from the Mendelian 3:1 segregation ratio (Table S2, see online supplementary material). To preliminarily map genes related to stem lodging, we conducted Mutmap analysis using the parents and two mixed DNA pools from the F_2_ population (Table S3, see online supplementary material). Based on the identified homozygous SNP loci, the frequency of allele gene SNPs was assessed by analysing DNA sequencing variations, and ΔSNP index values were obtained. A distinct peak on chromosome 8 was observed (126314622-165 820 095; approximately 39.50 Mb), indicating significant differences in the ΔSNP index (Fig. 2a). Further, a series of KASP (Competitive Allele-Specific PCR) markers were developed using SNPs information from the candidate region for genotyping (Table S1, see online supplementary material). Next, based on phenotypic data of individual plants from the F_2_ population, we performed genotyping to identify candidate region. The result indicated that the candidate region was located in SNP153164345 and SNP154539577 markers, with a physical distance of about 1.37 Mb. (Fig. 2b; Table S4, see online supplementary material).

Additionally, analysis of chromosomal structural variations between *slr1* and WT revealed a deletion spanning approximately 105 Kb (Chr08:153762245–153 867 356) within the candidate region of 1.37 Mb (Fig. 2c), covering five genes: CaZ8g15200, CaZ8g15210, CaZ8g15220, CaZ8g15230, and CaZ8g15240 (Fig. 2d). Based on the annotation information, CaZ8g15200 was identified as ARATH TF PIF3 (*Ca*PIF3), primarily involved in photomorphogenesis [69]; CaZ8g15210 as *fibrous sheath CABYR-binding protein* (*CaFSCB*); CaZ8g15220 as carbohydrate esterase (*CaCE*); CaZ8g15230 as a *hypothetical protein FXO38_26921* (*CaFXO*); and CaZ8g15240 as a *MYB61* TF (*CaMYB61*). Using cDNA from the WT and *slr1* stems as templates, the amplification of coding sequences from five genes detected the specific band only in the WT, but not in *slr1* (Fig. 2e). RT-qPCR analysis failed to detect any of the five genes in *slr1* (Fig. 2f). Therefore, PCR and RT-qPCR revealed a large-fragment deletion in *slr1*mutant.

### *CaSLR1* mediates stem lodging by affecting SCW thickness and stem strength in peppers and tomatoes

**Figure 3:**
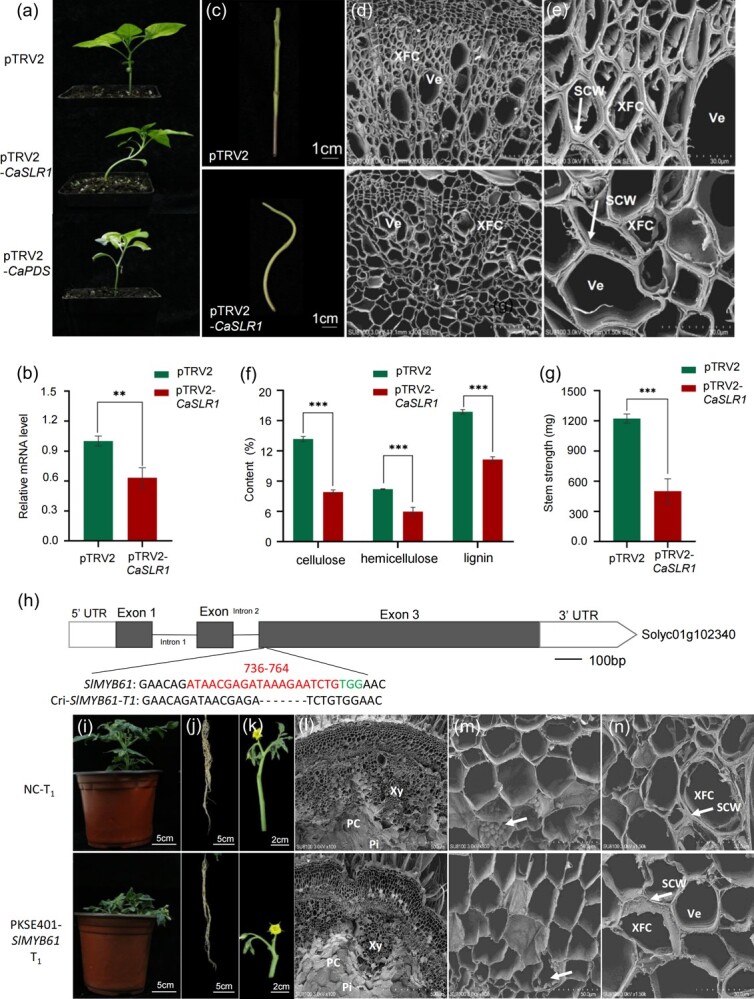
*CaSLR1* mediates stem lodging by affecting SCW thickness and stem strength in peppers and tomatoes. **(a)** Virus-induced gene silencing of *Ca*SLR1 in pepper, scale = 2.5 cm. **(b)** RT-qPCR analysis the relative expression levels of *CaSLR1* in the *CaSLR1*-silenced line (pTRV2-*CaSLR1*) and control (pTRV2, NC) lines. **(c)** The stems of *CaSLR1*-knockdown and control lines; scale = 1 cm. **(d)** The xylem tissues from the stems of the *CaSLR1*-knockdown and control lines, respectively; scale = 100 μm. **(e)** The secondary xylem from the stems of *CaSLR1*-knockdown and control lines, respectively; scale = 30 μm. **(f)** The contents of cellulose, hemicellulose, and lignin from the stems of the *CaSLR1*-knockdown and control lines, respectively. **(g)** The stem strength of *CaSLR1*-knockdown and control lines. The results were expressed as the mean ± SE (*n* = 3). ^**^P < 0.01 and ***P < 0.001, as determined by Student's *t*-test. **(h)***SlMBY61*, encoding a homolog of *Ca*SLR1, was edited at the third exon in tomato (Micro-Tom). PAM: protospacer-associated motif. Seven bases (TAAAGAA) were edited. Target bases: ATAACGAGATAAAGAATCTG. PAM: TGG. **(i)** The phenotype of T_1_ generation from the knockout line (Cri-*SlMYB61*) and no knockout line (negative control, NC). Scale = 5 cm. **(j)** The root from T_1_ generation. Scale = 5 cm. **(k)** The flower branches and inflorescences from T_1_ generation. Scale = 2 cm. **(l)** Scanning electron microscopy analysis of T_1_ generation stem. Scale = 500 μm. Xy: xylem; PC: parenchymal cell; Pi: pith. **(m)** The parenchymal cell closed to xylem of T_1_ generation stem. Scale = 50 μm. PC: parenchymal cell. The arrow points to the granular body. **(n)** The xylem fiber cell closed to secondary xylem from T_1_ generation stem. Scale = 50 μm. SCW: secondary cell wall; XFC: xylem fiber cell; Ve: vessel. The arrow points to SCW.

MYB61 has been identified as a regulator influencing cellulose and SCW formation [47]. Consequently, CaZ8g15240 was recognized as a crucial candidate gene and designated *CaSLR1*. Utilizing VIGS technology to silence *CaSLR1* in WT plants resulted in noticeable twisting and lodging characteristics in their stems (Fig. 3a). RT-qPCR analysis confirmed a significant down-regulation of *CaSLR1* expression in the stems, validating the successful silencing of *CaSLR1* (Fig. 3b). These findings elucidated the role of *CaSLR1* in mediating stem lodging development in pepper.

Given that SCW component biosynthesis affects plant mechanical support [3, 4], SEM analysis of the stems from *CaSLR1*-silenced and control (pTRV2) plants (Fig. 3c) revealed a denser mechanical structure in the xylem of control plants, whereas the mechanical tissue of the xylem in *CaSLR1*-silenced plants exhibited cell wall collapse (Fig. 3d). Particularly, the cell walls of xylem fiber cells in *CaSLR1*-silenced plants appeared thinner compared to controls (Fig. 3e), indicating that silencing *CaSLR1* leads to differences in xylem development. Moreover, the contents of the SCW components were significantly reduced (Fig. 3f). Stem strength analysis demonstrated that control plants exhibited a puncture strength of 1222.97 mg, whereas *CaSLR1*-silenced plants could withstand only 501.47 mg (Fig. 3g), indicating a reduction in stem strength due to the thinning of SCW.

**Figure 4:**
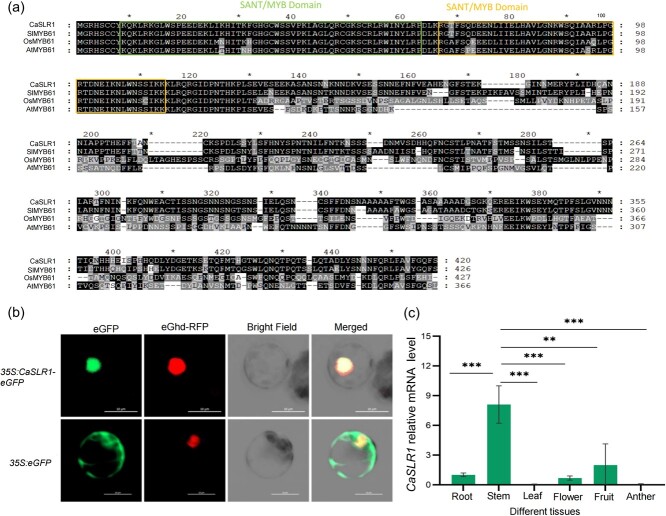
Subcellular localization and expression pattern of *Ca*SLR1. **(a)** Multiple sequences alignment of MYB61. MYB61 includes two SANT/MYB domains: 9–63 and 66–114. The *Ca*SLR1 from the cultivated pepper species *Capsicum annuum* L.; *Sl*MYB61 from *Solanum lycopersicum* L.; *Os*MYB61 from *Oryza sativa* L.; and *At*MYB61 from *Arabidopsis thaliana* L. **(b)** Subcellular localization of *Ca*SLR1. Red fluorescent protein (Ghd-RFP) was used as the nuclear label. Scale = 10 μm. **(c)** Relative expression levels of *CaSLR1* in the different tissues, roots, stems, leaves, blooms, fruits, and anthers of pepper plants. The results were expressed as the mean ± SE (*n* = 3). ^**^P < 0.01 and ^***^P < 0.001, as determined by Student's *t*-test.

To uncover the functional conservation of *Ca*SLR1, we used the CRISPR/Cas9 system to knock out *SlMYB61* (Solyc01g102340), a homolog of *CaSLR1*, in tomatoes. Sequencing of the PCR product from the *SlMYB61* knockout (Cri-*SlMYB61*) lines revealed a seven-base edit at the target site (Fig. 3h). The Cri-*SlMYB61* lines were self-crossed to obtain a stable genetic T_1_ generation, referred to as Cri-*SlMYB61*-T_1_. Compared to the wild-type (NC-T_1_), Cri-*SlMYB61*-T_1_ plants exhibited stem lodging (Fig. 3i). Additionally, the Cri-*SlMYB61*-T_1_ plants had fewer root branches (Fig. 3j) and shorter inflorescence branches (Fig. 3k) compared to NC-T_1_.

SEM analysis of cross-sectioned stems from NC-T_1_ and Cri-*SlMYB61*-T_1_ plants revealed significant differences. The pith (Pi) area in Cri-*SlMYB61*-T_1_ was enlarged compared to NC-T_1_ (Fig.3l), with stems from the latter containing abundant and well-developed granular plastids, while those from Cri-*SlMYB61*-T_1_ exhibited abnormal development (Fig. 3m). Moreover, the secondary cell walls (SCWs) of xylem fiber cells (XFCs) in NC-T_1_ were thicker, whereas those in Cri-*SlMYB61*-T_1_ were thinner (Fig. 3n), indicating that the edited *SlMYB61* affected stem structure, resulting in thinner SCWs in XFCs in tomatoes. These results suggest that *SlMYB61* knockout in tomatoes leads to alterations in plant structure, notably in stem strength and stability, indicating a conserved function of SLR1 in both peppers and tomatoes.

### Subcellular localization and expression pattern of *CaSLR1*

A preliminary exploration of the function of *Ca*SLR1, according to annotation information, revealed that *Ca*SLR1 belongs to a typical MYB family with 1796 bp in length, consisting of three exons and two introns, and with a 1263 bp long CDS, encoding 421 amino acids (Fig. S2a, see online supplementary material). *Ca*SLR1 (protein ID:XP_016538869) and *Sl*MYB61 (protein ID: XP_004230371) were identified to be the most closely related based on the comparative analysis of a phylogenetic tree. Interestingly, the homologous proteins of *Ca*SLR1 were identified from other species such as *Mus musculus*, *Saccharomyces cerevisiae S288C*, *Caenorhabditis elegans*, *Chlamydomonas reinhardtii*, *Oryza sativa*, *Nicotiana tabacum*, *A. thaliana* (Fig. S2b, see online supplementary material), indicating the evolutionary conservatism of SLR1. *Ca*PIF3*, Ca*FSCB*, Ca*CE*,* and *Ca*FXO are species-specific, lacking homologous genes in both animals and microorganisms (Fig. S3, see online supplementary material). Further, the sequence alignment analysis showed that two SANT/MYB domains in SLR1 were highly consistent between *C. annuum* (*Ca*SLR1) and *S. lycopersicum* (*Sl*MYB61), but they were slightly different among *C. annuum* (*Ca*SLR1), *O. sativa* (*Os*MYB61), and *A. thaliana* (*At*MYB61) (Fig. 4a), hinting at slightly different evolution.

To further elucidate the function of *Ca*SLR1, subcellular localization was examined by transiently transforming Arabidopsis protoplasts with the 35S:*CaSLR1*-*eGFP* expression vector. Confocal microscopy revealed GFP fluorescence distributed in the cell membrane and nucleus. Co-localization analysis with the nuclear marker Ghd-RFP confirmed the presence of *Ca*SLR1 in the nucleus (Fig. 4b), suggesting its role in gene regulation.

**Figure 5:**
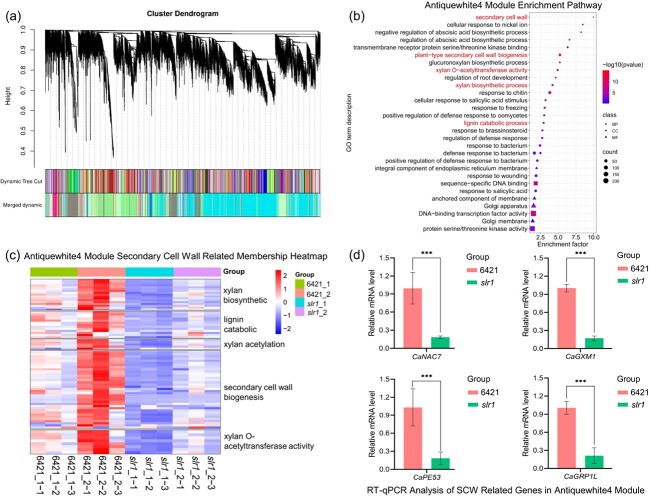
WGCNA analysis. **(a)** Genes cluster dendrogram analysis in which different gene modules are represented by branches and various colors. **(b)** GO enrichment analysis of the genes in the antiquewhite4 module. **(c)** The expression heatmap of SCW biosynthesis-related candidate genes in the antiquewhite4 module. **(d)** RT-qPCR analysis of four SCW biosynthesis-related genes in the antiquewhite4 module, *CaNAC7*: *NAC transcription factor 7*; *CaGXM1: glucuronoxylan 4-O-methyltransferase 1*; *CaPE53: pectinesterase 53*; and *CaGRP1L*: *glycine-rich cell wall structural protein 1-like*. The results were expressed as the mean ± SE (*n* = 3). ^***^P < 0.001, as determined by the Student's *t*-test.

To investigate the organ-specific expression patterns of *CaSLR1* in peppers, qRT-PCR analysis was conducted. The results revealed that the expression of *CaSLR1* was highest in the stems, with levels 8.01, 138.80, 11.97, 4.06, and 98.27-fold higher compared to the roots, leaves, flowers, fruits, and anthers, respectively, reaching significance (Fig. 4c). These results imply that *CaSLR1* participates in regulating stem development.

### RNA-seq analyses between WT and *slr1*

To delve into the molecular mechanisms underlying stem lodging in *slr1* mutants, RNA-seq was conducted using stem samples from 25- and 45-day-old WT and *slr1* mutants. After removing adaptor sequences, over 86% of clean reads were mapped to the reference genome ‘Zunla_1’, with a GC content of about 43% and a Q30 quality score exceeding 87% (Table S5, see online supplementary material). Differential expression analysis revealed 545 differentially expressed genes (DEGs) at 25 days and 1546 DEGs at 45 days in* slr1* mutants compared to WT. Notably, 251 DEGs were common between the two time points (Fig. S4a, see online supplementary material). The enrichment analysis of GO and KEGG uncovered that the DEGs were significantly enriched in pathways related to cell wall development, including plant-type SCW biogenesis and phenylpropanoid biosynthesis (Fig. S4b and c, Table S6, see online supplementary material). Protein–protein interaction (PPI) analysis showed that a regulatory network centered around *CaSLR1* was formed during stem development (Fig. S4d, see online supplementary material).

To explore the co-expression patterns of *CaSLR1*, WGCNA was performed. The Principal Component Analysis model indicates a high consistency among the three repetitions (Fig. S5a, see online supplementary material). WGCNA revealed that 30 branches were clustered (Fig. S5b and c, see online supplementary material). Notably, the antiquewhite4 module, which encompassed the candidate gene *CaSLR1* (Zunla_1 reference genomic gene ID: Capana08g001690), was delineated (Fig. 5a; Table S7, see online supplementary material). GO enrichment unveiled significant enrichment of genes within the antiquewhite4 module in various cellular components, particularly those related to SCW formation, plant-type SCW biogenesis, xylan O-acetyltransferase activity, and xylan biosynthetic process (Fig. 5b; Table S8, see online supplementary material). This highlights the potential role of *CaSLR1* in orchestrating these crucial processes during stem development.

The expression patterns of the SCW biosynthesis-related genes within the antiquewhite4 module, such as c*ellulose synthase A catalytic subunit*, *β-1,4-xylosyltransferase IRX10L*, *UDP-glucuronate: xylan α-glucuronosyltransferase*, and *fasciclin-like arabinogalactan protein* exhibited consistent down-regulation during stem development in *slr1*, particularly evident after stem lodging in *slr1* plants (the 45-day-old plants; *slr1*_2) (Fig. 5c; Table S9, see online supplementary material). This suggests a correlation between stem lodging and the biosynthesis of cell wall components in pepper. RT-qPCR confirmed the downregulation of cell wall formation-related genes from the antiquewhite4 module, including *NAC transcription factor 7* (*CaNAC7*), *glucuronoxylan 4-O-methyltransferase 1* (*CaGXM1*), *pectinesterase 53* (*CaPE53*), and *glycine-rich cell wall structural protein 1-like* (*CaGRP1L*), in stems of 45-day-old *slr1* mutants (*slr1*_2), affirming the reliability of the results (Fig. 5d).

### *Ca*NAC6 binds to the promoter of *CaSLR1* to affect the SCW biosynthetic pathway

**Figure 6:**
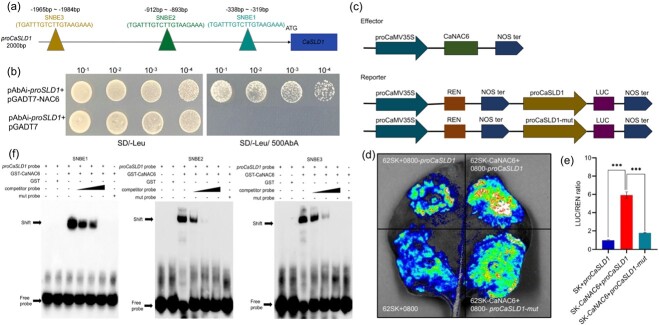
*Ca*NAC6 binds to the promoter of *CaSLR1* and induces its transcription. **(a)** The position of the three secondary wall NAC binding elements (SNBEs) in the 2000 bp long promoter of *CaSLR1*. **(b)** The potential binding of *Ca*NAC6 with *CaSLR1* promoter in yeast. SD/−Leu, medium lacking leucine. SD/−Leu/500 AbA, medium lacking leucine and added with 500 ng/mL aureobasidin A (AbA). **(c)** Schematic of the vector used for the Dual-luciferase reporter system. LUC, firefly luciferase. REN, renilla luciferase. **(d)** LUC images of the tobacco leaves after transient infiltration. **(e)** The ratio of LUC to REN activity. The results were expressed as the mean ± SE (*n* = 6). ^***^P < 0.001, as determined by the Student's *t*-test. **(f)** The binding of *Ca*NAC6 with the SNBE elements of *CaSLR1* was determined using an EMSA assay.

NAC and MYB transcription factors (TFs) are recognized as the ‘master switches’ controlling SCW formation [8]. Within the antiquewhite4 module, a *Ca*NAC6 (Capana10g001138) TF was found to be co-expressed with *CaSLR1* (Fig. S4d and Table S7, see online supplementary material). NAC29, a homologous protein of *Ca*NAC6, has been implicated in regulating SCW deposition in rice [48]. NAC TFs contribute to SCW biosynthesis by binding to SNBE elements that existed in MYB promoters [12]. We identified three SNBEs elements within 2000 bp promoter in *CaSLR1* (Fig. 6a).

To confirm the binding between *Ca*NAC6 and the *CaSLR1* promoter, a Y1H assay was conducted. Yeast cells were co-transformed with pAbAi-*proSLR1* along with either pGADT7-*NAC6* or pGADT7 vectors (NC) and cultured on SD/−Leu medium supplemented with 500 ng/mL of AbA. The results showed that the co-transformed pGADT7-*NAC6* and pAbAi-*proSLR1* were able to promote yeast cells' growth on the AbA selective medium, unlike the negative control cells (Fig. 6b).

Further, to confirm whether *Ca*NAC6 could regulate *CaSLR1* expression, we injected with 62SK-*CaNAC6* and *proSLR1*-LUC into tobacco leaves via the LUC system. Compared to the negative control (62SK and *proSLR1*-LUC), the ratio of LUC to REN was 5.92-fold higher. However, substituting *proSLR1*-LUC with *proSLR1*-mut-LUC resulted in a decreased ratio to 1.79, indicating a significant reduction in gene transcriptional activation (Fig. 6c–e). To demonstrate that *Ca*NAC6 was directly bound to the three SNBE elements of *CaSLR1*, an EMSA assay was conducted using purified *Ca*NAC6 recombinant protein fused with a GST tag (Fig. S6, see online supplementary material). The finding revealed that *Ca*NAC6 was bound to the biotin-labeled SNBE1, 2, and 3 probes, with binding strength inversely proportional to the concentrations of competitor probes. However, mutation of the SNBEs core sequence abolished the binding, indicating the specificity of the interaction (Fig. 6f). Our results suggest that *Ca*NAC6 positively regulated the expression of *CaSLR1*.

### *CaSLR1* regulates genes expression associated with SCW formation pathways in pepper stems

To further explore the underlying molecular mechanism of *CaSLR1*-mediated stem development in pepper, RNA-seq was used to analyse RNA samples extracted from stems of 45-day-old *CaSLR1*-silenced and control plants (pTRV2). After filtering out adaptor sequences, more than 87% clean reads were obtained, which were then mapped to the pepper ‘Zunla_1’ reference genome. The primary quality Q30 was above 92.9% (Fig. S10, see online supplementary material). In total, we identified 2105 DEGs, including 1132 up-regulated and 973 down-regulated genes (Fig. S7, see online supplementary material). The GO analysis revealed significant enrichment of DEGs in the cellular components associated with the plant-type cell wall and cellulose synthase complex. In terms of biological processes, enrichment was observed in cell wall organization, phenylpropanoid metabolism, and cellulose microfibril organization, indicating that *CaSLR1*-silencing affected the expression of genes related to cell wall components (Table S11, see online supplementary material).

To investigate the synergistic effects of DEGs in signal transduction and biochemical metabolism pathways, we performed KEGG enrichment analysis. The results revealed that DEGs were mainly enriched in 17 pathways, including hormone signal transduction, starch and sucrose metabolism, phenylpropanoid biosynthesis, etc. (Table S12, see online supplementary material), indicating that *CaSLR1* affected lignin biosynthesis. Research indicates that the protein encoded by the auxin pathway gene ARF binds to the promoter regions of essential genes associated with lignin biosynthesis, including *caffeoyl-CoA O-methyltransferase* (*CCoAOMT2*) or *4-coumarate: coenzyme A ligase* (*4CL3*, *4CL7*, and *4CL9*), and activates their expression thereby affecting mechanical bending in bamboo [29].

**Figure 7:**
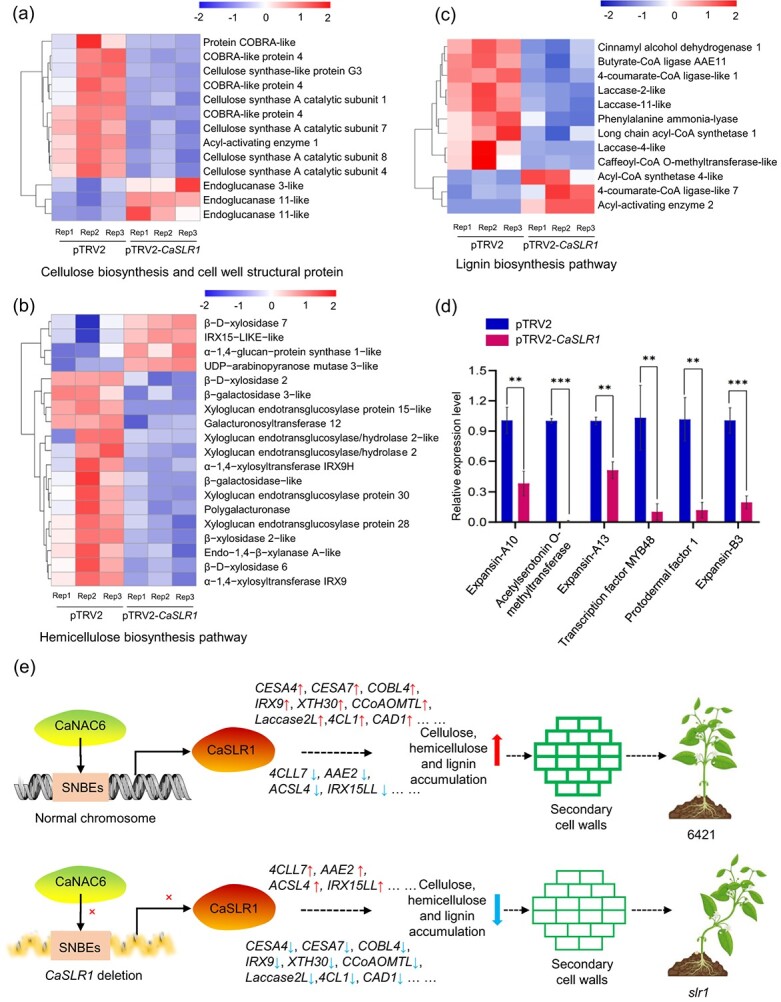
*CaSLR1* regulates the expression of cellulose, hemicellulose, and lignin biosynthesis related genes in pepper stems. **(a)** The expression-related heatmap of genes involved in the cellulose biosynthesis pathway and cell wall structural proteins. **(b)** The expression-related heatmap of genes involved in the hemicellulose biosynthesis pathway. **(c)** The expression-related heatmap of genes involved in the lignin biosynthesis pathway. **(d)** qRT-PCR analysis verified the down-regulated genes related to SCW formation pathways using RNA-seq. The results were expressed as the mean ± SE (*n* = 3). **P < 0.01; ***P < 0.001, as determined by the Student's *t*-test. **(e)** A hypothesis suggests that *CaSLR1* regulated the stem lodging resistance. In WT, *Ca*NAC6 binds to SNBE elements in the promoter of *CaSLR1*, thereby inducing its transcription. As a result, the stem accumulated cellulose, hemicellulose, and lignin, which promotes the deposition of the SCW and increases stem strength, enabling the stem remain upright. In contrast, in the mutant *slr1*, a deletion of *CaSLR1* and its promoter abolished the binding of *Ca*NAC6 to the SNBEs. The transcription of *CaSLR1* was inhibited, and the accumulation of cellulose, hemicellulose, and lignin in stems was reduced. Accordingly, it resulted in the thinning of the SCW, weakening of stem strength, and finally, stem lodging. ‘×’ marked that the effect was cancelled. *CESA*: *cellulose synthase*; *COBL4*: *COBRA-like 4*; *IRX9*: *irregular xylem 9*; *XTH30*: *xyloglucan endotransglucosylase protein 30*; *CCoAOMTL*: *caffeoyl-CoA O-methyltransferase 1ike*; *laccase 2 L*: *laccase 2-like*; *4CL1*: *4-coumarate-Co-A ligase 1*; *CAD1*: *cinnamyl alcohol dehydrogenase 1*; *4CCL7*: *4-coumarate-Co-A ligase like 7*; *AAE2*: *Acyl-activating enzyme 2*; *ACSL4*: *Long chain acyl-CoA synthetase 4-like*; *IRX15LL*: *IRX15 − LIKE − like*.

Additionally, we explored the gene expression of cellulose, cell wall structural proteins, and hemicellulose biosynthesis pathways in *CaSLR1* silenced plants. Heatmap analysis revealed a significant down-regulation in numerous genes associated with cell wall structural protein biosynthesis, such as COBRA-like (COBL) and cellulose synthase A catalytic subunit (CESA) (Fig. 7a). Similarly, genes related to hemicellulose biosynthesis, including *IRX9H*, *IRX9*, and *xyloglucan endotransglucosylase protein 30* (*XTH30*), were also down-regulated (Fig. 7b). *XTH30* is recognized for its crucial role in building and restructuring xyloglucan cross-links [49]. Notably, the expression of genes essential for lignin biosynthesis, including *caffeoyl-CoA O-methyltransferase-like* (*CCoAOMTL*), *laccase-4-like*, *laccase 2-like*, *laccase 11-like*, *4-coumarate-CoA ligase-like 1* (*4CL1*), and *cinnamyl alcohol dehydrogenase 1* (*CAD1*) was also down-regulated (Fig. 7c), indicating that *CaSLR1* may play a role in regulating genes expression associated with lignin biosynthesis. Furthermore, the down-regulated genes involved with cell wall formation were verified by qRT-PCR (Fig. 7d). This was consistent between the qRT-PCR results and transcriptomic sequencing data, validating the reliability of our findings. Therefore, we propose that *CaSLR1* modulates the expression of secondary cell wall (SCW) biosynthesis genes, thereby influencing stem development in pepper.

## Discussion

Stems serve as the primary load-bearing structure in plants, providing resistance against lodging. However, during stem development, cells within the vascular bundles, fibers, and vessels gradually lose their protoplasm, leaving only the cell walls to offer mechanical support [7]. The mechanical strength and stability of the stem are largely influenced by cell wall components [50, 51]. Here, a typical pepper lodging mutant *slr1*, induced by EMS, exhibited constrictive xylem and defective secondary cell wall development, accompanied by a significant decrease in cellulose, hemicellulose, and lignin contents (Fig. 1c–h). Via fine mapping and chromosome structure variation analysis, a deletion region of approximately 105 Kb on chromosome No. 8 was identified as the candidate region, including CaZ8g15200, CaZ8g15210, CaZ8g15220, CaZ8g15230, and CaZ8g15240 five genes (Fig. 2b–c). Among five genes, CaZ8g15230 was not expressed in WT and *slr1* mutants. Hence, we only focus on another four genes. CaZ8g15210 encodes carbohydrate esterase. Carbohydrate esterase family members have been suggested to aid in reducing cell wall rigidity by cleaving covalent linkages between lignin and glucuronoxylan [52]. CaZ8g15240 encodes the transcription factor MYB61. Previous research has demonstrated that *Pb*MYB308 binds with *Pb*MYB61 to negatively regulate the synthesis of stone cell lignin in pear fruits [53]. In *A. thaliana*, the *AtMYB61* mutant exhibited reduced xylem formation and impaired xylem cell structure [54]. Our results reveal that *Ca*SLR1 (CaZ8g15240) is homologous to *At*MYB61 (Fig. S2b, see online supplementary material). Given that *CaSLR1* knockdown lines exhibit a lodging phenotype similar to *slr1*, it is considered a candidate causal gene. In the study, notably, the F_2_ population did not conform to the expected Mendelian 3:1 separation ratio, and only one peak was observed in the Mutmap analysis (Fig. 2a). Previous research indicated that germination and seedling establishment were compromised in *Arabidopsis myb61* mutants [55]. Our findings revealed that the *CaSLR1* mutation significantly decreased the number of fruits and the seed germination percentage. Specifically, the seed germination rate was 99% in WT but only approximately 52% in *slr1* mutants (Fig. S1a–c, see online supplementary material). We speculate that *Caslr1* affects fruit and seed development, potentially explaining the abnormal F_2_ segregation ratio. Additionally, the *slr1* mutant is caused by a substantial mutation. Contrary to the predominant point mutations induced by EMS, as Xiong *et al.* [56] reported, the genomic mutagenesis library confirmed that EMS can also cause large fragment deletions [57]. For instance, 870 large homozygous deletions were detected in tetraploid wheat and 7971 in hexaploid wheat [58], likely a consequence of using higher EMS dosage.

The softness of stems relies on the elastic behavior of fibers, which is determined by both the overall load and the composition of the SCW. Overexpressing plants with p1300-SND1P-XND1 exhibited recumbent stems, while stem length remained largely unaffected [59]. Alterations in SCW formation and components within vascular cells have been shown to affect stem mechanical properties [34, 35, 60]. Our study reveals that the cellulose content of *CaSLR1*-knockdown lines is significantly reduced by 36.39% compared to pTRV2 controls (Fig. 3f). While both *CaSLR1* and *AtMYB61* influenced cellulose content, notable differences in stem erectness were observed between peppers and *Arabidopsis*. *CaSLR1*-knockdown lines displayed complete lodging with a prostrate posture (Fig. 3a). However, the erectness of the inflorescence stems of the *Atmyb61* mutant was not affected [54]. In rice lines where *OsMYB61* was overexpressed, cellulose content increased, while total lignin and xylose (one of the main components of hemicellulose) contents remained unchanged at the basal internodes [47]. In our study, *CaMYB61*-knockdown lines exhibited reductions in hemicellulose and lignin contents. Additionally, the stem strength of *CaMYB61*-silenced lines decreased by approximately 59%. The differing effects of MYB61 on the pathways of hemicellulose and lignin biosynthesis in various species could be a significant factor contributing to the alterations observed in stem development.

Furthermore, MYB61, a crucial multifunctional protein, influences various metabolic processes in plant growth. *OsMYB61* is directly regulated by Growth Regulator Factor 4 (GRF4), a known regulator of nitrogen using efficiency (NUE), which promotes biomass production in rice [61]. In this study, biomass decreased in *CaSLR1*-silenced pepper lines and *SlMYB61* knockout tomato lines (Fig. 3i–3k; Fig. S1f, see online supplementary material). As a regulator of resource allocation, *AtMYB61* expression was observed in the metabolic sink, particularly in the xylem and roots. Mutants of *AtMYB61* exhibited reductions in lateral roots and xylem vessels [54]. In *slr1* mutant and *CaSLR1*-knockdown lines, the decreased contents of cellulose, hemicellulose, and lignin revealed that the *CaSLR1* affected the formation of structural carbohydrates in plants. These findings support the conclusion that MYB61 influences resource allocation. Furthermore, despite the homology between *CaSLR1* and *AtMYB61*, there are notable differences in their phenotypic effects. These distinctions may arise from variations in stem types between pepper and *Arabidopsis* inflorescences, as well as differences in amino acids sequences within conserved domains (Fig. 4a). Another contributing factor could be the variations in types of mutations, which lead to varying phenotypic outcomes. In this study, the deletion of the entire *CaSLR1* gene exhibited a more pronounced effect (Fig. 1a), in contrast to the subtler phenotypes observed in *AtMYB61*-knockout lines in *Arabidopsis* [54]. An intriguing avenue for future research is to investigate whether mutations in different regions of *CaSLR1* would impact the biosynthesis of cell wall components, thereby influencing the deposition of SCWs and potentially causing plant-specific variations.

A complex multilevel transcriptional network in plants acts on the biosynthetic genes of SCW components, thereby regulating the thickening process of SCWs [62]. Studies on *Arabidopsis* mutants have established a clear consensus that NAC and MYB TFs act as switches in regulating SCW biosynthesis [62, 63]. MYB46 was one of the earliest discovered SCW master switches [64]. The down-regulation of MYB46 and MYB83 drastically repressed SCW thickening in both vessels and fibers, thereby causing collapsed vessels and reduced plant growth [65]. SND1 binds to the SNBE element in MYB46 promoter and activates its expression [11]. In this study, WGCNA indicated that *CaSLR1* and *CaNAC6* clustered in the same module (Fig. 5a). *Ca*NAC6 exhibited homology to NAC29, which regulated SCW deposition in rice [48]. Overexpressing *OsNAC29* lines exhibited thicker internodes and significantly increased cellulose content [47]. Our study revealed that *Ca*NAC6 specifically binds to SNBE elements in *CaSLR1* promoter, thereby activating its expression (Fig. 6b–d). Therefore, *Ca*NAC6 and *Ca*SLR1 are important regulators of SCW formation in pepper.

Secondary wall-associated NACs TFs activate the secondary MYB master switch, leading to the expression of downstream SCW formation-related genes [63]. In our study, *CaSLR1* regulated the expression of downstream genes involved in cellulose, hemicellulose, and lignin biosynthesis, including *CESA*, *COBL4*, *IRX9*, *XTH30*, *CCoAOMTL, laccase 2 L*, *4CL1*, *CAD1*, *4CCL7*, *IRX15LL* (Fig. 7a–c). On the Golgi apparatus, CESAs assemble into cellulose synthase complexes, which are then transported to the plasma membrane for cellulose synthesis, ultimately depositing cellulose on one side of the cell wall [66]. The spatial control of lignin chemistry depends on different combinations of laccases with nonredundant activities immobilized in cell wall layers [67]. Given all this, *CaSLR1* and *CaNAC6* may be crucial master switches in the molecular network of regulating secondary cell wall formation in peppers. Based on our study, a hypothesis was proposed to elucidate the mechanism of stem lodging resistance in pepper. In the WT, *Ca*NAC6 binds to the SNBEs in the promoter of *CaSLR1*, inducing the transcription of *CaSLR1*. Consequently, the expression of *CaSLR1* promoted the accumulation of cellulose, hemicellulose, and lignin, resulting in thickened secondary cell walls (SCWs) and erect stems. Conversely, in the *slr1* mutant, the complete deletion of *CaSLR1* and its promoter prevents *Ca*NAC6 from binding to the SNBE elements, reducing the accumulation of these structural components, leading to thinner SCW and stem lodging (Fig. 7e). These findings highlight the *Ca*NAC6-*Ca*SLR1 module that contributes to lodging resistance, underscoring the pivotal role of *Ca*SLR1 within the regulatory network for lodging resistance.

## Materials and methods

### Plant materials and growth conditions

The elite inbred line ‘6421’, which serves as a foundational breeding stock extensively utilized across China, was treated with 1% EMS (medial lethal dosage) to construct a mutant library [18]. We had sown 10 000 mutants and 200 WT seeds, and then the individual plant was conducted self-cross. Next, we harvested the self-cross seeds from individual plants to construct the M1 generation, and selected M2 generation with stem lodging from M1 self-cross seeds. Using the same planting screening method as the M2 generation, we obtained a genetically stable stem lodging mutant *slr1* M3 generation [19]. WT and *slr1* plants were planted in a greenhouse with 65% relative humidity. Various tissues were gathered from the growing plants for gene expression analysis, including roots, stems, leaves, blooms, fruits, and anthers.

### Scanning electron microscopy (SEM) and determination of stem components

Fresh tissues were collected, taking care to minimize mechanical damage, such as bruising, pulling, and squeezing. Based on a previously described method [35], the tissue blocks were placed in electron microscopy fixative for 2 h and then preserved at 4°C for transportation. The tissues were observed with an SEM, and images were taken.

The 45-day-old stems from WT, *slr1*, pTRV2, and pTRV2-*CaSLR1* lines were collected. Cellulose, hemicellulose, and lignin contents were determined using kits (Cellulose content kit product code: G0715W, hemicellulose content kit product code: G0716W, lignin content kit product code: G0708W48, Suzhou Grace Biotechnology Co. Ltd, Suzhou, China).

### Candidate gene mapping

We identified the chromosome region of candidate target genes using MutMap method [21]. Leaf samples were taken from 30 of the WT and *slr1* plants of the F_2_ segregating population. We extracted the genomic DNA (DNA extraction kit, Product code: DP360, Tiangen Biotech Co. Ltd, Beijing, China) [22] of 30 upright and 30 lodging plants from the F_2_ population, respectively. Next, we constructed two mixed DNA pools and performed the whole-genome resequencing on the HiSeq™ PE150 platform (Illumina, Inc., San Diego, CA, USA). Reads data were filtered by Fastp [23], and clean reads were mapped to the reference of ‘Zhangshugang’ (http://ted.bti.cornell.edu/ftp/pepper/genome /Zhangshugang/) genome [20] using bwa mem v0.7.12 [24]. Duplicated reads derived from PCR were marked with samblaster. The generated BAM file was utilized to identify single nucleotide polymorphisms (SNPs) and insertions/deletions (InDels) using GATK [25]. Structural variations were identified by manta [26]. SNPs were filtered using bcftools [27] with the parameters QD < 2.0 || MQRankSum < −12.5 || ReadPosRankSum < −8.0. The SNP-index value was calculated to locate the traits, with the wild-type (WT) parent selected as the reference parent to compute the SNP-index of the two offspring.

To fine-map the candidate genes, we detected SNP co-segregation via the Kompetitive Allele-Specific PCR (KASP) genotyping technique. Based on a previously described method [28], we designed allele-specific primers based on the 200 bp sequence upstream and downstream of the SNPs, shown in Table S1 (see online supplementary material). A total of 483 F_2_ individuals were used for KASP genotyping. The SNP genotyping detection system PARMS (penta-primer amplification refractory mutation system) (Gentides Biotech Co., Ltd) was used according to the manufacturer's protocol. The primers were synthesized by the Gentides Biotech Co. Ltd (Wuhan, China). A 10 μl reaction system was prepared, including 5uL 2× PARMS (containing fluorescent universal primers FAM, HEX), 0.7uL specific amplification primers, 1uL (50 ng) DNA template and 3.3 uL double distilled H_2_O. Signals were detected using the LightCycle® 96 Real-Time PCR system (Roche, Basel, Switzerland) [28].

### Phylogenetic tree construction and subcellular localization of *CaSLR1*

MAFFT v7.475 was used [30] for multiple *Ca*SLR1 protein sequence alignment. Then, the default parameters and maximum likelihood of the IQ-TREE v2.1.2 software was used to construct the phylogenetic tree [31] with the bootstrap value set as 1000. Finally, Itol (https://itol.embl.de/) was used to visualize the results.

Using One Step Cloning Kit (Product code: C112–02, Vazyme Biotech Co. Ltd, Nanjing, China), as cited by Liu *et al.* [32], we insert the coding sequence (CDS) of *CaSLR1* into the pYBA1132 expression vector to construct the pYBA1132-*CaSLR1*-GFP vector. The leaves of the 3-week-old *Arabidopsis* ‘Columbia’ plants were used for protoplast isolation. The pYBA1132-*CaSLR1*-GFP and the empty PYBA1132-GFP vectors were proliferated and extracted. They were then used to co-transform *Arabidopsis* protoplasts with a nuclear marker (Ghd-RFP) and cultured under low light conditions for 8–10 h. Fluorescence was observed and captured via an LSM800 laser scanning confocal microscope (Zeiss, Germany).

### RT-qPCR analysis

Total mRNA was extracted using the TRIzol reagent (Product code:ET121–01, TransGen Biotech), and 2 μg of total RNA was reversed into cDNA via a reverse transcription kit (Product code: AG11706, Accurate Biology [Hunan] Co. Ltd, Changsha, China). RT-qPCR was implemented using SYBR Green (Product code: AG11701) [33] on a LightCycle® 96 Real-Time PCR system (Roche, Basel, Switzerland). The actin gene expression, as an internal control, in pepper was normalized.

### Virus-induced gene silencing (VIGS)

To decrease the expression of target genes in the WT, we conducted virus-induced gene silencing following a previously established method with slight modifications [35]. We identified specific regions of *CaSLR1* via online tool SGN VIGS (https://vigs.solgenomics.net/) [36, 37]. Unique primers were used to amplify the cDNA of pepper stems, yielding a product of approximately 300 base pairs. Next, we used the One Step Cloning Kit (Product code: C112–02) [32] to clone the fragment into the pTRV2 vector, which has been double-digested with *EcoRI* (Product code: FD0274) and *BamHI* (Product code: FD0054) (Thermo Fisher Scientific, Waltham, Massachusetts, USA). The vectors were then transformed into *Agrobacterium* strain GV3101. The whole cotyledons of ten-day-old seedlings were infected by this *Agrobacterium* strain GV3101. After six weeks, we collected the first and second internode stems above the cotyledons for scanning electron microscope analysis, and determined the contents of cellulose, lignin, and hemicellulose in same samples. The stem strength of the first internode was measured eight weeks post-infection.

### CRISPR/Cas9-mediated *Ca*SLR1 homolog encoding gene *SlMY61* knockout in tomato

The homologous sequence of *Sl*MYB61 (Solyc01g102340) was obtained by aligning *Ca*SLR1 to the tomato genome (*Solanum lycopersicum*). Based on the CDS of *SlMYB61*, we designed CRISPR target sites with the assistance of the CRISPR-P 2.0 website [16]. Two pairs of PCR primers for two CRISPR targets ‘TTGAACTTCATGCAGTTCTT’ and ‘ATAACGAGATAAAGAATCTG’ were designed (Table S1, see online supplementary material). Next, the PGTR plasmid served as a template for PCR, and the product was cloned into the CRISPR expression vector pKSE401 by Golden Gate cloning [68]. The vector was used to transform *Agrobacterium tumefaciens* EHA105 cells, constructing pKSE401-expressing strain Cri-*SlMYB61* for inoculation. Tomato (Micro Tom) cotyledons were transformed using a previously established method [38].

### RNA-seq analysis

Total mRNA was extracted from the stems of the WT, *slr1*, pTRV2, and pTRV2-Ca*SLR1* lines using TRIzol reagent (Product code: ET121–01, TransGen Biotech Co., Ltd, Beijing, China) in three biological replicates from each group. Upon assessing the sample quality, we constructed the library and subsequently sequenced on the Novaseq platform [39]. For RNA-seq processing, Fastp v0.20.0 was employed to filter the raw data, removing low-quality reads and adaptor sequences to obtain high-quality clean data [40]. The clean reads were mapped to the pepper genome (Zunla_1, NCBI genome code: 4072) [41] using HISAT2 v2.1.0 [42]. Next, StringTie v2.2.3b [43] was utilized to compare the transcript assembly and quantify gene expression in the dataset. DESeq2 [44] was used to quantitatively determine the differentially expressed genes (DEGs) with the criteria |log2FC| ≥ 1 and FDR ≤ 0.05 [45]. Based on the GO and KEGG databases, the DEGs were annotated via BLASTALL v2.2.26 (https://ftp.ncbi.nlm.nih.gov/blast/executables/blast+/LATEST/) with an e-value set to 1e-5.

### Weighted gene co-expression network analysis (WGCNA)

Based on gene expression levels, we constructed a co-expression network using the WGCNA package in R. Firstly, we normalized expression data to FPKM +1 value and then transformed it to log2. The correlation between module genes was subsequently analysed using WGCNA [28]. The WGCNA hierarchical network was utilized to construct a co-expressed gene network module. BLASTALL v2.2.26 (https://ftp.ncbi.nlm.nih.gov/blast/executables/blast+/LATEST/), with an e-value set to 1e-5, was used to annotate the module genes via GO and KEGG databases and Perl scripts.

### Yeast one-hybrid assay (Y1H)

To explore the interactions of *Ca*NAC6 with *CaMYB61,* Y1H assays were conducted. The ORFs of *CaNAC6* were cloned into the pGADT7 backbone as prey [46]. A tandem of three SNBEs of the *CaMYB61* promoter was cloned into the pABAi backbone as bait. Both vectors were transformed into Y1H Gold yeast strain cells using Clontech's small-scale transformation method. Glass beads were used to coat SD/−Leu /AbA until no bacterial liquid flow was observed. Samples were then cultured on plates at 30°C in an incubator for 5–7 days.

### Dual luciferase reporter analysis (LUC)

To validate the activation of *CaMYB61* by *Ca*NAC6, the coding sequence (CDS) of *Ca*NAC6 was cloned into the pGreenII 62-SK backbone to construct an effector vector. The promoter sequence of *CaMYB61* was then inserted into the pGreenII 0800-LUC (luciferase) backbone to drive the expression of LUC gene, creating a reporter vector. These vectors were transformed into GV3101 cells, which were subsequently injected into tobacco leaves and kept in the dark. After 2–3 days, we used a 5200 Imaging System (Tanon Science & Technology, Shanghai, China) to capture the fluorescence signal on leaves. Subsequently, we used a Dual-Luciferase® Reporter Assay System (Product code: E1910, Promega, Madison, WI, USA) to measure the fluorescence values of LUC and REN (Renilla luciferase). Each sample underwent testing with a minimum of six biological replicates.

### Electrophoretic mobility shift analysis (EMSA)

The CDS of *CaNAC6* was inserted into the PGX-6P-1 GST backbone and then transformed into *Escherichia coli* BL21 (DE3). Expression induction resulted in the production of the NAC6-GST fusion protein. Following the manufacturer's instructions, the LightShift chemiluminescence EMSA kit (Product code: Bes5003, Biotechnology Co., Ltd, Guangzhou, China) was utilized to detect the interaction between *Ca*NAC6 and the SNBE of the *CaSLR1* promoter. The synthesized SNBE element was annealed with a biotin-labeled double-stranded DNA probe (Novagen Biotechnology Co., Ltd, Beijing, China). The mutant biotin-labeled and unlabeled probes were incubated with the NAC6-GST fusion protein to assess its binding capability. Following the binding reaction, the complex underwent electrophoresis on a 6% acrylamide gel and was subsequently transferred to a nylon membrane. After rinsing, the nylon was positioned in a membrane box, and the X-ray film was exposed for 5 minutes. Gel shift was facilitated using Anti-streptavidin-HRP (horseradish peroxidase) conjugated antibody (Product code: HRP-66001, Reda Henghui Technology Development Co. Ltd, Beijing, China).

### Statistical analysis

The experimental data were analysed using Microsoft Excel 2013. A histogram was generated using GraphPad Prism v7.04. Statistical significance was evaluated using Student's *t*-test. All qRT-PCR (quantitative real-time-PCR) analyses were carried out with at least three replicates.

## Supplementary Material

Web_Material_uhae169
