# Supplementary material for: Stem lodging Resistance-1 controls stem strength by positively regulating the biosynthesis of cell wall components in Capsicum annuum L
Source: Hortic Res. 2024 Jun 20;11(8):uhae169. doi: 10.1093/hr/uhae169 (PMC11317896; doi:10.1093/hr/uhae169)

## Supplementary Figure legends

**Fig. S1 Comparied the phenotypes between wild-type (WT) and *slr1* mutants.** (a) Phenotypes at different stem developmental stages. (b) Fruits during the red ripening period, scale = 8 cm. (c) Seed germination scene on five days, scale = 1.5 cm. (d) Seed germination percentage rate. (e) Above-ground weight. (f) Fresh root weight. (g) Total fresh weight. (h) First internode length. (i) Ratio of leaf length and leaf width. The results were expressed as mean ± s.e.m (n = 3). ***, P <0.001, n.s, not significant, as determined by Student's-*t* test.

**Fig. S2 Gene structure schematic diagram and phylogenetic tree of *Ca*SLR1.** (a) Schematic diagram of *CaSLR1* gene structure. (b) Phylogenetic tree of SLR1. The black pentagram marks the identified proteins in this study.

**Fig. S3 Phylogenetic tree of PIF3, FSCB, CE, and FXO.** (a) Phylogenetic tree of PIF3. (b) Phylogenetic tree of FSCB. (c) Phylogenetic tree of CE. (d) Phylogenetic tree of FXO. The red pentagram marks the identified proteins in this study.

**Fig. S4 RNA-seq analysis between WT and *slr1* mutants.** (a) Differential expression genes (DEGs) analysis from WT and *slr1* at different stem development stages. (b) Gene Ontology (GO) enrichment analysis of DEGs. (c) Kyoto Encyclopedia of Genes and Genomes (KEGG) enrichment analysis of DEGs. (d) Protein-protein interaction (PPI) networks of CaSLR1.

**Fig. S5 Weighted gene co-expression network analysis (WGCNA).** (a) The repeatability of samples was detected by principal component analysis (PCA). (b) Soft threshold analysis in WGCNA. (c) Expression patterns of genes in antiquewhite4 module in different samples. (d) Heat map of MYB and NAC transcription factors in antiquewhite4 module.

**Fig. S6 SDS-PAGE analysis of the purified recombinant GST-NAC6 protein.** M: molecular mass standard; Line1: supernatant after cell lysis; Line2: cell lysis precipitates; Line3: perforating fluid; Line4: 20mM GSH elution 1; Line5: 20mM GSH elution 2; Line6: 20mM GSH elution 3. The arrow indicates the target protein GST-NAC6.

**Fig. S7 Differential expression genes (DEGs) between pTRV2 and pTRV2-*CaSLR1* plants.**

Supplementary Fig. S1

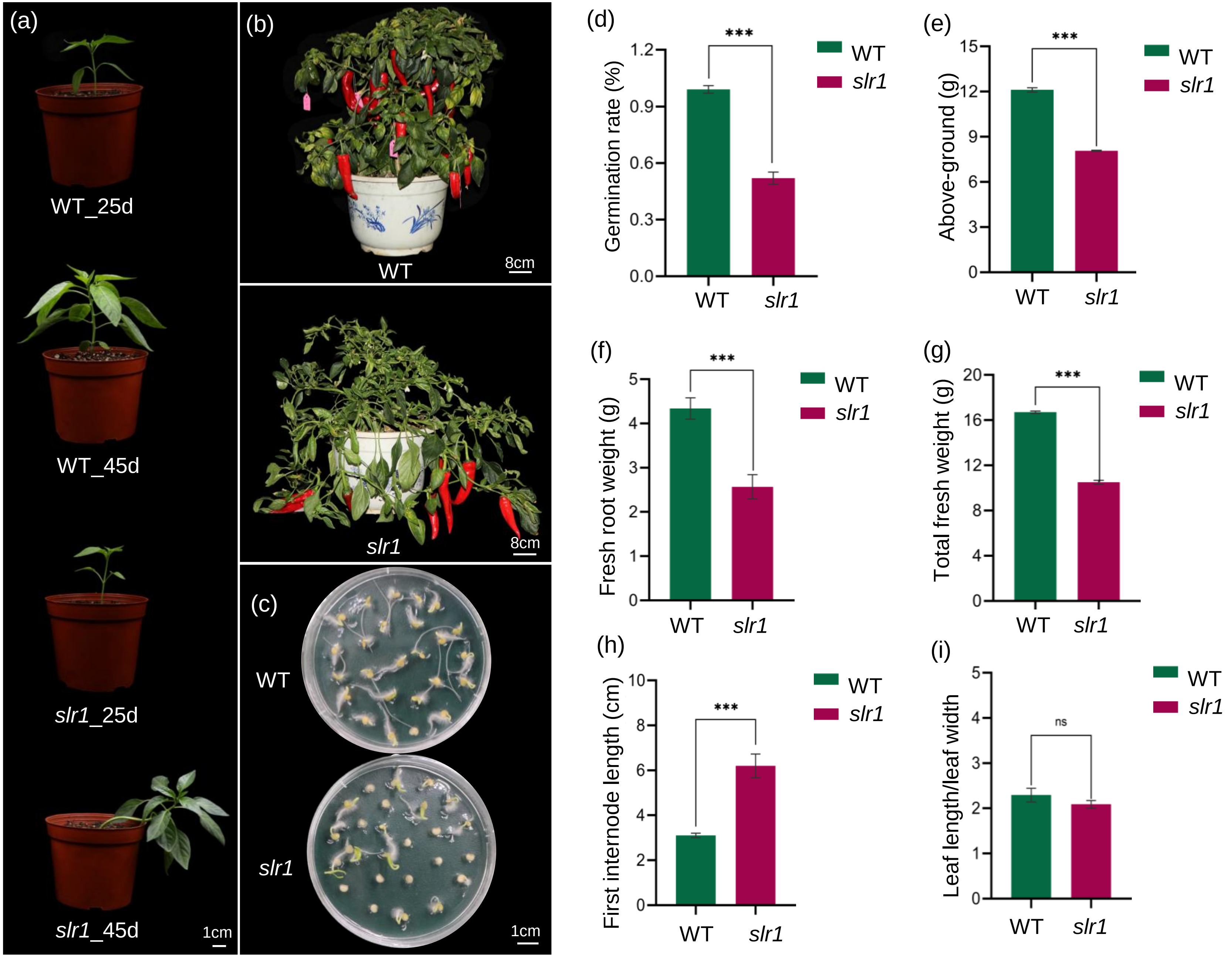

# Supplementary Fig. S2

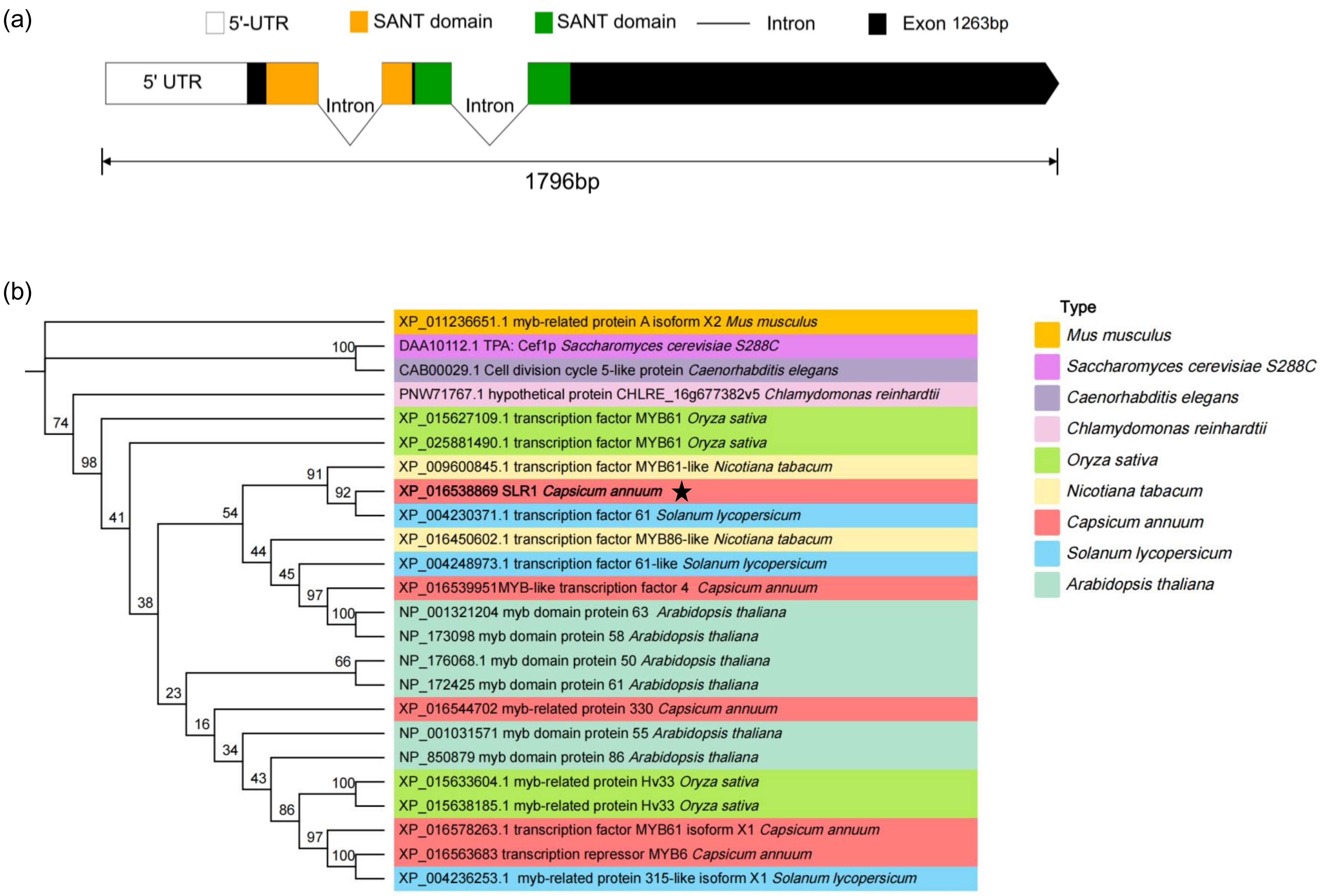

Supplementary Fig. S3

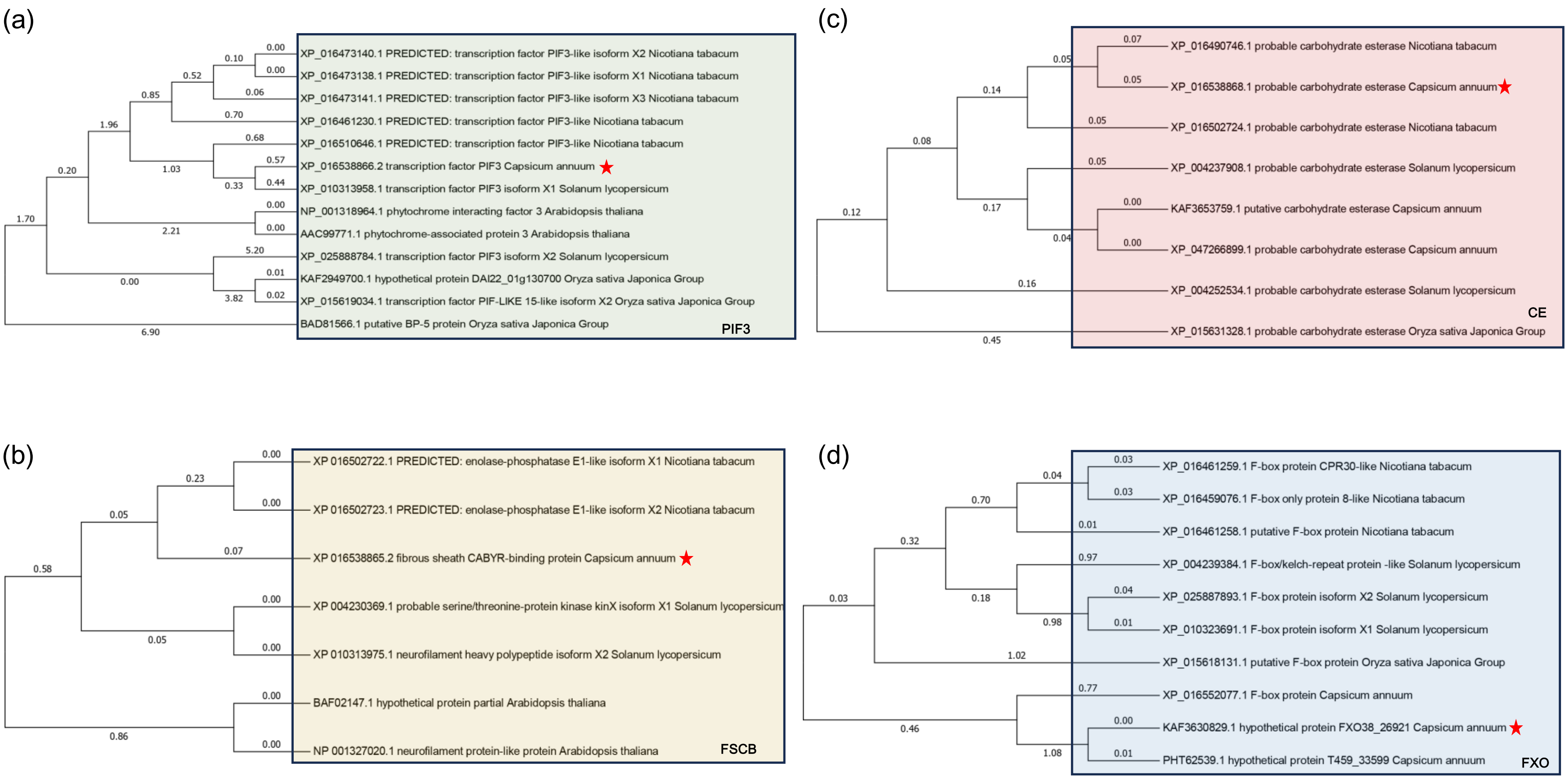

Supplementary Fig. S4

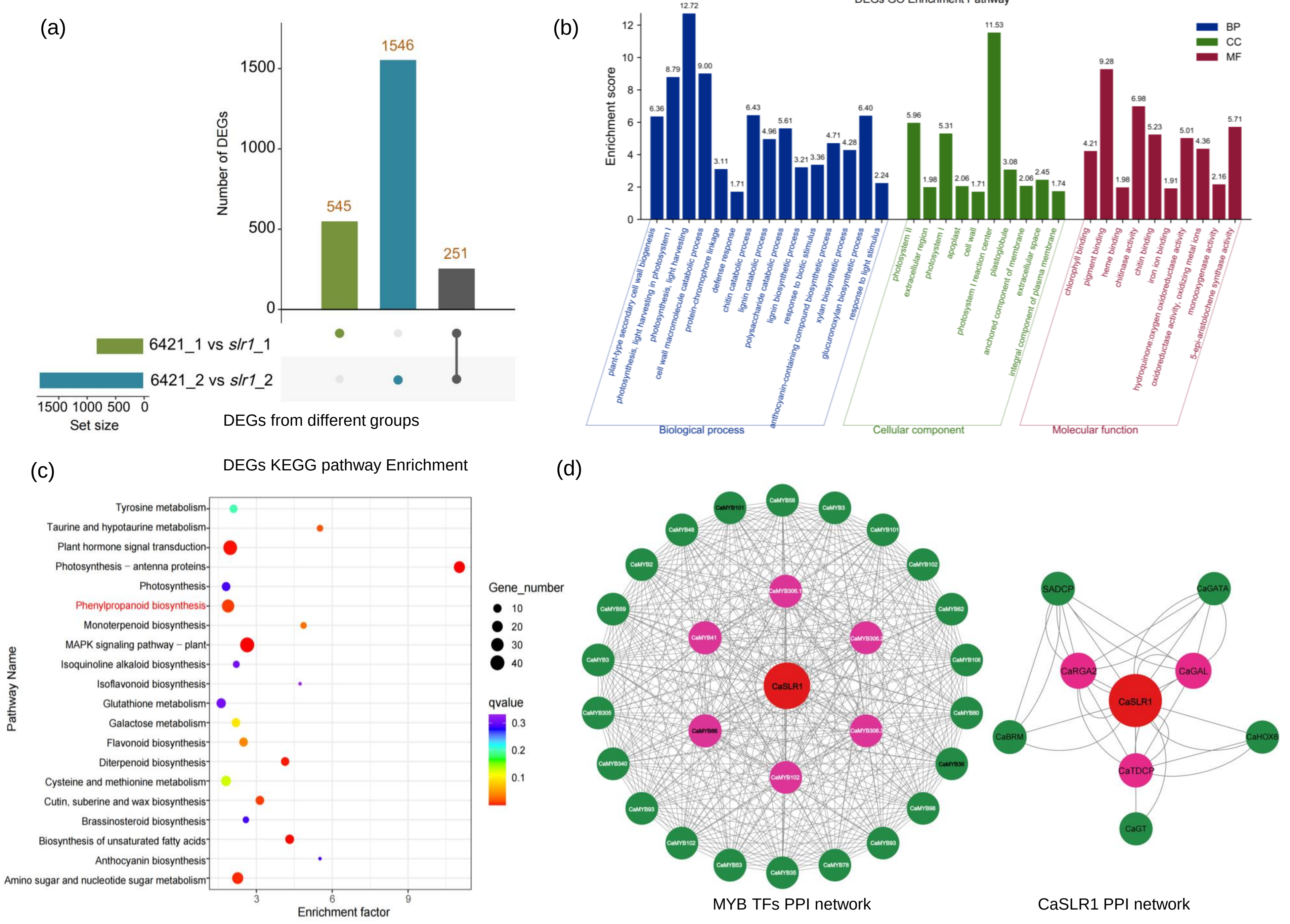

Supplementary Fig. S5

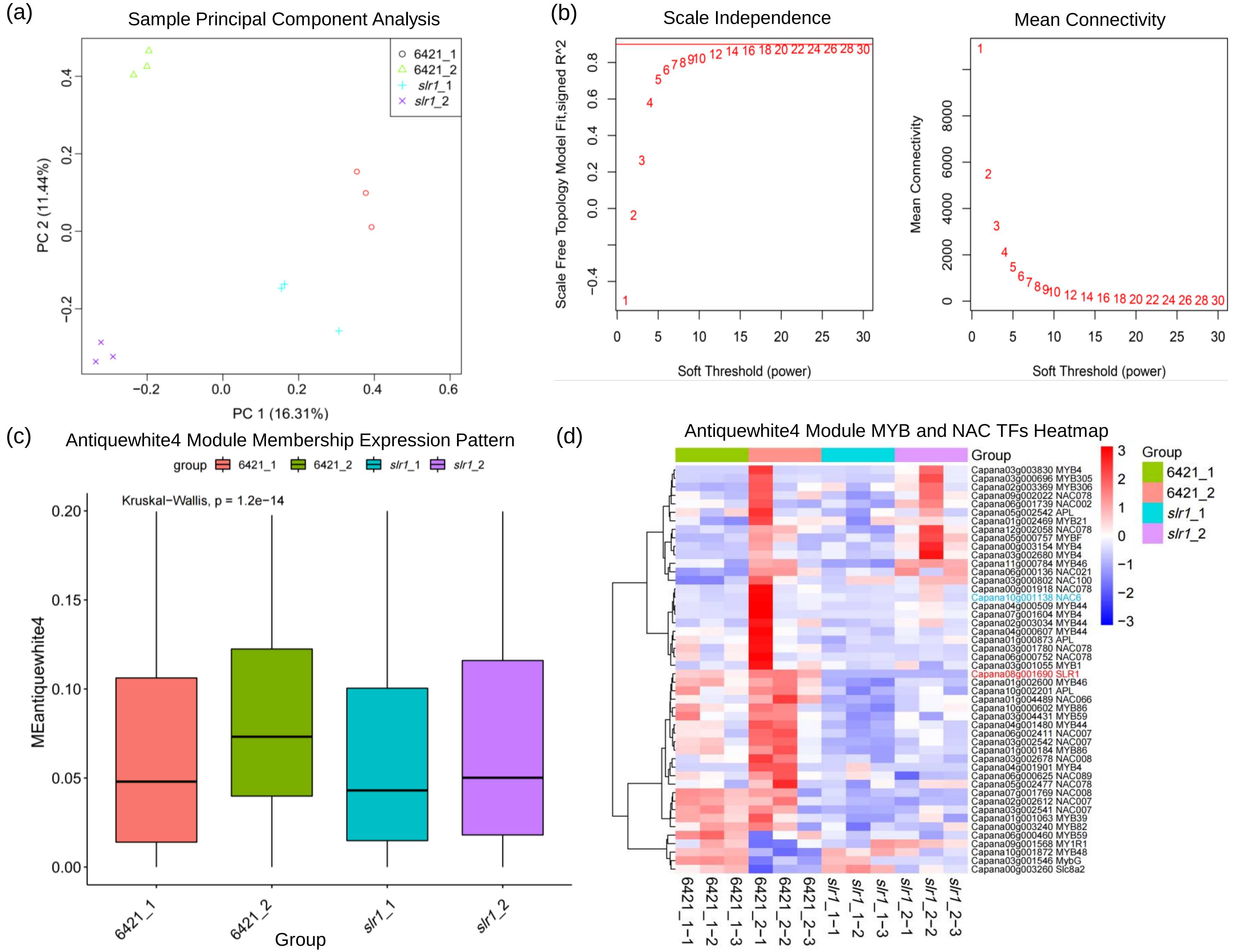

# Supplementary Fig. S6

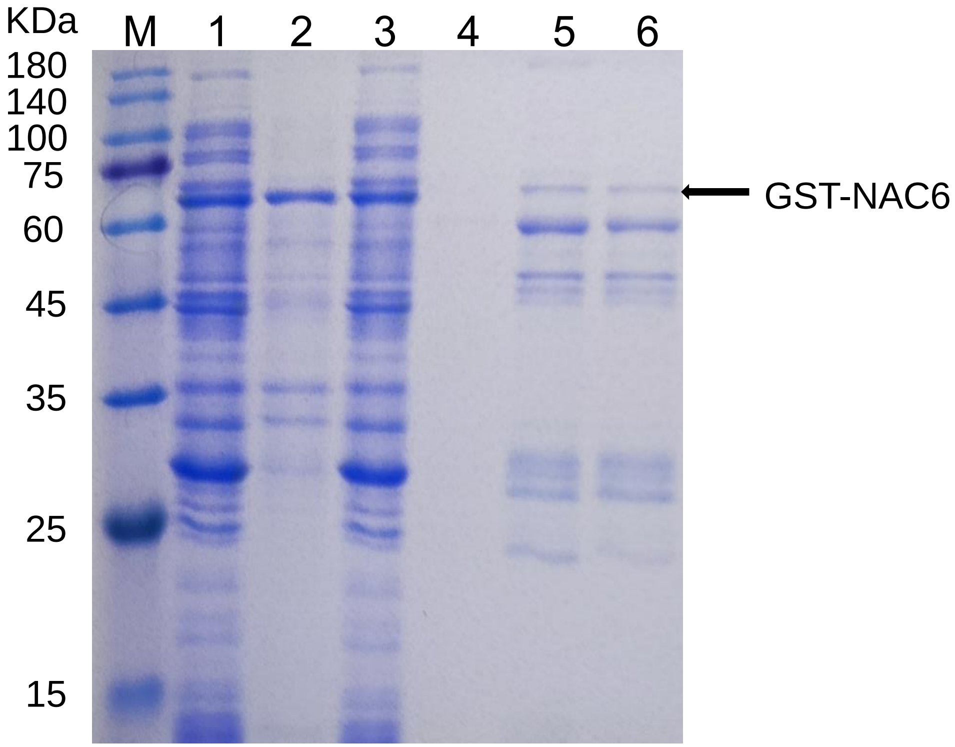

Supplementary Fig. S7

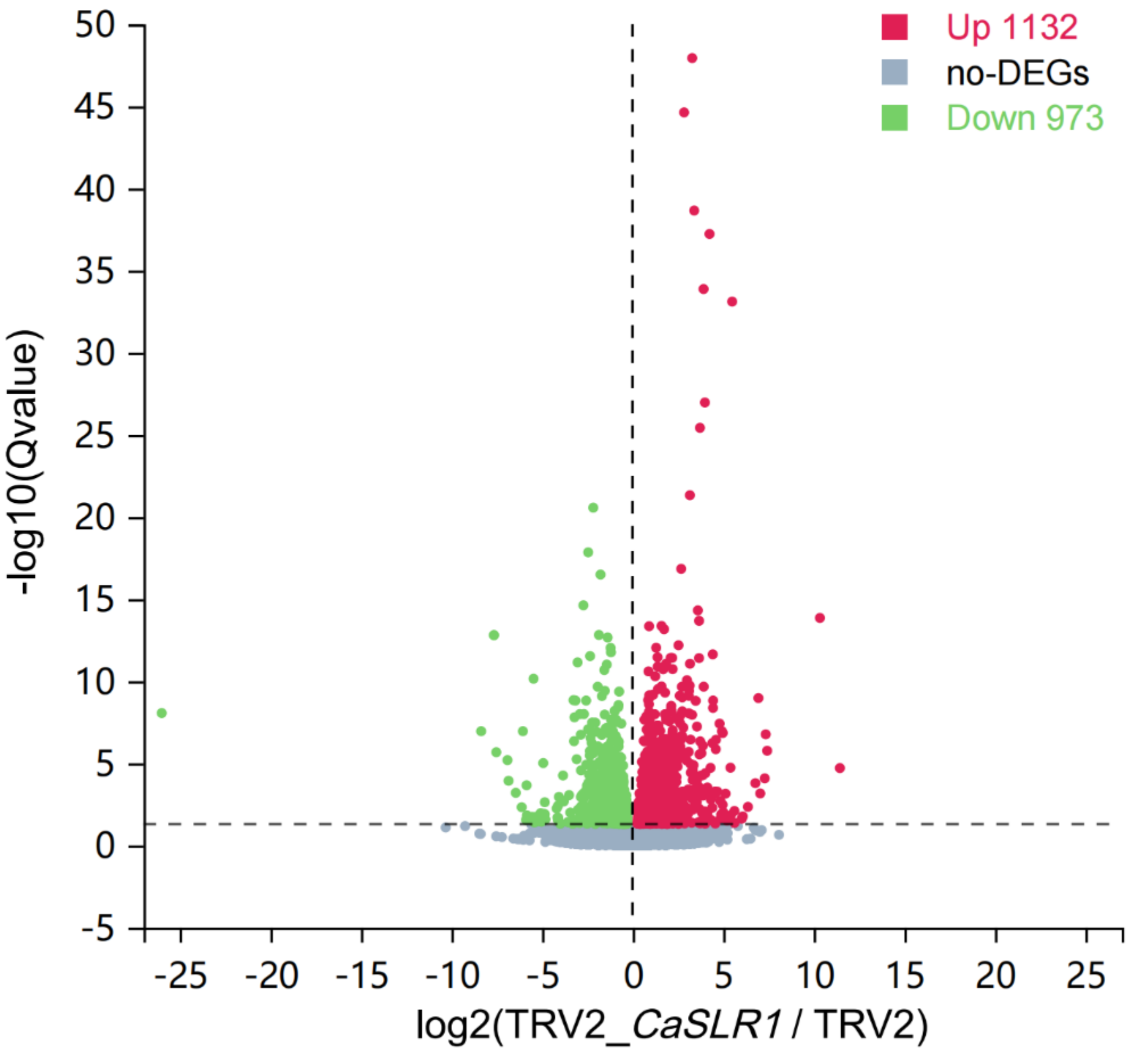

Supplement: Web_Material_uhae169 [file web_material_uhae169.zip › Supplementary Figures20240507.pdf]
